# Apitherapy and Periodontal Disease: Insights into In Vitro, In Vivo, and Clinical Studies

**DOI:** 10.3390/antiox11050823

**Published:** 2022-04-22

**Authors:** Manoj Kumar, Suraj Prakash, Radha, José M. Lorenzo, Deepak Chandran, Sangram Dhumal, Abhijit Dey, Marisennayya Senapathy, Nadeem Rais, Surinder Singh, Phillip Kalkreuter, Rahul D. Damale, Suman Natta, Marthandan Vishvanathan, Sangeetha Kizhakkumkara Sathyaseelan, Sureshkumar Rajalingam, Sabareeshwari Viswanathan, Yasodha Murugesan, Muthamilselvan Muthukumar, Aravind Jayaraman, Murugasridevi Kalirajan, Samy Selim, Ryszard Amarowicz, Mohamed Mekhemar

**Affiliations:** 1 Chemical and Biochemical Processing Division, ICAR–Central Institute for Research on Cotton Technology, Mumbai 400019, India; 2 School of Biological and Environmental Sciences, Shoolini University of Biotechnology and Management 3 Sciences, Solan 173229, India; surajpandiar75@gmail.com (S.P.); radhuchauhan7002@gmail.com (R.); 3 Centro Tecnológico de la Carne de Galicia, rúa Galicia n° 4, Parque Tecnológico de Galicia, San Cibrao das Viñas, 32900 Ourense, Spain; jmlorenzo@ceteca.net; 4 Área de Tecnología de los Alimentos, Facultad de Ciencias de Ourense, Universidad de Vigo, 32004 Ourense, Spain; 5 Department of Veterinary Sciences and Animal Husbandry, Amrita School of Agricultural Sciences, Amrita Vishwa Vidyapeetham University, Coimbatore 642109, India; c_deepak@cb.amrita.edu; 6 Division of Horticulture, RCSM College of Agriculture, Kolhapur 416004, India; sdhumal@msu.edu; 7 Department of Life Sciences, Presidency University, 86/1 College Street, Kolkata 700073, India; abhijit.dbs@presiuniv.ac.in; 8 Department of Rural Development and Agricultural Extension, College of Agriculture, Wolaita Sodo University, Wolaita Sodo, Ethiopia; drsenapathy@wsu.edu.et; 9 Department of Pharmacy, Bhagwant University, Ajmer 305004, India; drx.nadeemrais@gmail.com; 10 Dr. S. S. Bhatnagar University Institute of Chemical Engineering and Technology, Panjab University, Chandigarh 160014, India; ssbhinder@pu.ac.in; 11 Dr. Seiler und Kollegen Oral Surgery, 70794 Filderstadt, Germany; phillipkalkreuter@gmail.com; 12 ICAR—National Research Centre on Pomegranate, Solapur 413255, India; rahul.damale@icar.gov.in; 13 ICAR—National Research Centre for Orchids, Pakyong 737106, India; nattabiochem@gmail.com; 14 Department of Seed Science and Technology, Amrita School of Agricultural Sciences, Amrita Vishwa Vidyapeetham University, Coimbatore 642109, India; v_marthandan@cb.amrita.edu; 15 Department of Plantation Crops and Spices, Kerala Agricultural University, Vellanikkara, Thrissur 680656, Kerala, India; sangeetha.ks@kau.in; 16 Department of Agronomy, Amrita School of Agricultural Sciences, Amrita Vishwa Vidyapeetham University, Coimbatore 642109, India; r_sureshkumar@cb.amrita.edu; 17 Department of Soil Science and Agricultural Chemistry, Amrita School of Agricultural Sciences, Amrita Vishwa Vidyapeetham University, Coimbatore 642109, India; v_sabareeshwari@cb.amrita.edu; 18 School of Agriculture and Biosciences, Karunya Institute of Technology and Sciences, Coimbatore 641114, India; yasoagri@gmail.com; 19 Department of Agricultural Entomology, SRM College of Agricultural Sciences, SRM Institute of Science and Technology, Chengalpattu 603201, India; muthukum2@srmist.edu.in; 20 Department of Agricultural Entomology, Amrita School of Agricultural Sciences, Amrita Vishwa Vidyapeetham University, Coimbatore 642109, India; j_aravind@cb.amrita.edu (A.J.); k_murugasridevi@cb.amrita.edu (M.K.); 21 Department of Clinical Laboratory Sciences, College of Applied Medical Sciences, Jouf University, Sakaka 72341, Saudi Arabia; sabdulsalam@ju.edu.sa; 22 Institute of Animal Reproduction and Food Research, Polish Academy of Sciences, Tuwima 10 Str., 10-748 Olsztyn, Poland; amaro@pan.olsztyn.pl; 23 Clinic for Conservative Dentistry and Periodontology, School of Dental Medicine, Christian-Albrecht’s University, 24105 Kiel, Germany

**Keywords:** apitherapy, propolis, periodontitis, gingivitis, oral bacterial disease, oral health

## Abstract

Periodontal diseases are caused mainly by inflammation of the gums and bones surrounding the teeth or by dysbiosis of the oral microbiome, and the Global Burden of Disease study (2019) reported that periodontal disease affects 20–50% of the global population. In recent years, more preference has been given to natural therapies compared to synthetic drugs in the treatment of periodontal disease, and several oral care products, such as toothpaste, mouthwash, and dentifrices, have been developed comprising honeybee products, such as propolis, honey, royal jelly, and purified bee venom. In this study, we systematically reviewed the literature on the treatment of periodontitis using honeybee products. A literature search was performed using various databases, including PubMed, Web of Science, ScienceDirect, Scopus, clinicaltrials.gov, and Google Scholar. A total of 31 studies were reviewed using eligibility criteria published between January 2016 and December 2021. In vitro, in vivo, and clinical studies (randomized clinical trials) were included. Based on the results of these studies, honeybee products, such as propolis and purified bee venom, were concluded to be effective and safe for use in the treatment of periodontitis mainly due to their antimicrobial and anti-inflammatory activities. However, to obtain reliable results from randomized clinical trials assessing the effectiveness of honeybee products in periodontal treatment with long-term follow-up, a broader sample size and assessment of various clinical parameters are needed.

## 1. Introduction

According to the World Health Organization (WHO), a study conducted by the Global Burden of diseases (2019) estimated that 3.5 billion people were affected by oral disease globally which includes different conditions such as oral cancer (causes approximately 180,000 deaths each year), periodontal disease, dental caries of primary teeth (nearly 520 million children suffer each year), dental caries of permanent teeth (2 billion people), birth defects such as cleft palate, and the oral manifestation of the human immunodeficiency virus (HIV) [1,2]. The risk factors include unhealthy diets (high in sugar), tobacco use, and alcohol consumption. However, in recent years, more interest in the adoption of favorable oral health behavior has been observed, which can facilitate better oral health [3,4,5]. In the last two years, 77% of countries have reported complete or partial disruption of oral health services due to the COVID-19 pandemic [2]. Some recent studies reported that risk factors for causing severe acute respiratory syndrome coronavirus 2 (SARS-CoV-2) infection might be associated with dental biofilms, especially dental plaque (subgingival), in patients with periodontitis [6]. Studies have also reported indirect and direct mechanisms for the association between poor oral health and the severity of COVID-19. The indirect mechanism relates to bacterial superinfections and inflammatory pathways, and the direct mechanism relates to angiotensin-converting enzyme II receptors (ACE-2) [6,7,8].

According to a Global Burden of Diseases study (2019), among different oral diseases, periodontal disease affects 20–50% of the worldwide population [1]. Periodontitis is caused by an abnormal host response from dental plaque to bacteria and affects teeth supporting structures, such as periodontal ligament, alveolar bone, and root cementum, resulting in tooth loss in some cases [9]. The increase in experimental evidence and clinical studies show a relationship between periodontal disease and systematic diseases, including Alzheimer’s disease, diabetes, cancers, and atherosclerosis [10,11,12,13,14,15]. Immunological research on periodontitis revealed the importance of the local host immune response in the pathogenesis of the periodontal disease [16]. The periodontal disease can be identified by soft periodontal tissue inflammation. The most common type of periodontal disease is gingivitis, which is mostly widespread at all ages. Almost all forms of periodontal disease are reported to be specific chronic bacterial infections caused by the overgrowth of a limited number of species in dental plaque, such as *Bacteroides forsythus*, *Treponema denticola*, and *Porphyromonas gingivalis* [9,17,18]. Meta-transcriptomics and metagenomic studies revealed that in the pathogenesis of periodontitis, a complex microbial community is involved instead of some specific periodontopathic bacteria [19,20,21,22,23]. Microorganisms present in dental plaque are the main cause of periodontitis, and the progression or severity of periodontitis is determined by the local host immune response [24,25]. The characteristics of periodontal disease include an increase in the peripheral polymorphonuclear oxidative response and inflammatory infiltrate presence. In the early phase of periodontitis, studies show that neutrophils arrive at the site of inflammation and kill pathogens using degranulation and phagocytosis [26,27]. In some cases, in the lateral stage of periodontitis, neutrophils become hyperactive, which increases proinflammatory cytokines, superoxides, and destructive enzymes that cause tissue destruction [24,28]. With the increase in reactive oxygen species (ROS) or the number of free radicals, oxidative stress is triggered and causes oxidative damage to the alveolar bone, periodontal ligament, and gingival tissue [29,30,31]. ROS causes the release of proinflammatory cytokines (i.e., tumor necrosis factor-α; interleukin-2, -6, and -8; and interferon-β), which play important roles in the pathogenesis of periodontal diseases [9,32].

In recent years, attention has grown towards the use of natural therapies given the numerous advantages offered by natural products compared to synthetic drugs [33,34,35]. Currently, more preference is given to natural therapies, such as apitherapy, due to the high safety margin, lower cost, and broad bioactivity compared to synthetic medicine [33]. Apitherapy is defined as the science and art of holistic healing and treatment from honeybees and their products, such as royal jelly, propolis, honey, and bee venom [36]. Royal jelly is produced from a combination of pollen and honey and contains essential fatty acids, nutrients, and vitamins, including A, B, C, D, and E [37,38]. Royal jelly is reported to prevent cell damage in HIV and cancer patients, lower blood cholesterol levels, aid in wound healing, and exhibit antimicrobial effects [39,40,41,42]. Purified bee venom is composed of enzymes and several active peptides, including apamin, melittin, mast cell-degranulating peptides, and adolapin, and is a natural toxin produced by *Apis mellifera* L. (honeybees) [43,44]. Bee venom is reported to treat back pain, rheumatoid arthritis (inflammatory disease), and skin diseases and acts as an anticancer agent in the treatment of breast cancer cells, prostate cancer cells, and lung cancer cells [45,46]. A recent study reported that bee venom was effective in periodontitis treatment, showing anti-periodontitis and anti-inflammatory effects [47].

Propolis is a natural nontoxic resinous compound produced by bees, and its composition includes 10% aromatic and essential oils, 10% pollen and other organic compounds, 30% waxes, and 50% vegetable resins [48,49]. The composition depends on various factors, such as bee species, geographical origin, and botanical origin, and the main component consists of phenolic esters, such as caffeic acid phenethyl ester and flavonoids [50,51]. Propolis is reported to have various bioactivities, such as antioxidant, anticancer, antimicrobial, anti-inflammatory, and anti-fungal activities [9,51,52]. Several in vitro and animal studies have suggested that propolis exhibits potential antioxidant effects. Propolis and its compound pinocembrin upregulate the enzymatic antioxidant pathway and induce Nrf-2 translocation to the nucleus following the expression of ARE-mediated antioxidant genes, including γ-GCS and HO-1. Propolis also regulates the expression of protein and mRNA of other antioxidant markers, including TrxR1, GCLC, LOX-1, GCLM, and γ-GCS [53,54,55]. Some recent studies reported that propolis and its compounds, such as CAPE, rutin, and myricetin inhibit ACE 2 receptors (essential for SARS-CoV-2 virus entry). This activity might be helpful in reducing the risk of COVID-19 complications [6,56,57]. Some studies also reported that propolis is effective in inhibiting periodontal pathogens, including *Prevotella intermedia* and *P. gingivalis*, and preventing alveolar bone loss in a periodontitis rat model in vivo [9]. Mouthwash, toothpaste, and dentifrices containing bee products, such as propolis and honey, have shown excellent effects in preventing gingivitis, tooth decay, periodontitis, and biofilm reduction [58,59,60].

In the last five years, more clinical trials and experimental evidence have been published indicating an increase in the trend towards the use of natural therapies with pharmacological activity in the treatment of various “oral bacterial diseases” and to provide better oral health. Given the anti-inflammatory, antimicrobial, and antioxidant activities of bee products, the practical application of bee products in dentistry might be helpful in the treatment of “oral bacterial disease” such as periodontitis, dental caries, and gingivitis. The main objective of this systematic review is to provide insight into the role of apitherapy in the treatment of periodontal disease. However, in vivo and clinical studies assessing the effects of bee products in the treatment of periodontal diseases are limited.

## 2. Materials and Methods

In the current review, the study selection process was conducted according to the guidelines of ‘Preferred Reporting Items for Systematic Reviews and Meta Analyses’ (PRISMA 2020) for systematic reviews [61].

### 2.1. Search Strategy

Various in vitro, in vivo, and clinical studies related to the role of apitherapy in the treatment of periodontal diseases were reviewed. An electronic literature search was performed using the PubMed, Web of Science, ScienceDirect, Scopus, clinicaltrials.gov, and Google Scholar databases. The following medical subject heading (MeSH) words were used individually in the search: honey, apitherapy, propolis, periodontitis, gingivitis, royal jelly, bee venom, and periodontal diseases. The following MeSH terms were used in combination: apitherapy and periodontal disease, honey and periodontal disease, propolis and periodontal disease, royal jelly and periodontal disease, bee venom and periodontal disease, propolis and gingivitis treatment, honey and gingivitis treatment, royal jelly and gingivitis treatment, and bee venom and gingivitis treatment. In the current study, an electronic literature search was performed to identify studies published within the period of 2016–2021 and were selected based on eligibility criteria, i.e., inclusion and exclusion criteria.

### 2.2. Inclusion and Exclusion Criteria

In the current analysis, studies were selected for review based on the following exclusion and inclusion criteria.

Exclusion criteria:

(i) Studies that did not have full text available.
(ii) Clinical trials that do not follow ethical guidelines.
(iii) Published studies in local languages except for English.
(iv) Nonrelevant studies (apitherapy in the treatment of other oral pathologies).
(v) Systematic reviews.

Inclusion criteria:

(i) In vitro, in vivo, and clinical studies evaluating the efficiency of honey, propolis, and royal jelly in the treatment of periodontal diseases.
(ii) Findings published in English.
(iii) Findings published within the period from 2016 to 2021.
(iv) Randomized and nonrandomized clinical trials.

After the selection of in vivo, in vitro, and clinical studies, data related to various bioactivities of honey and its compounds in periodontal disease treatment were collected.

**Figure 1:**
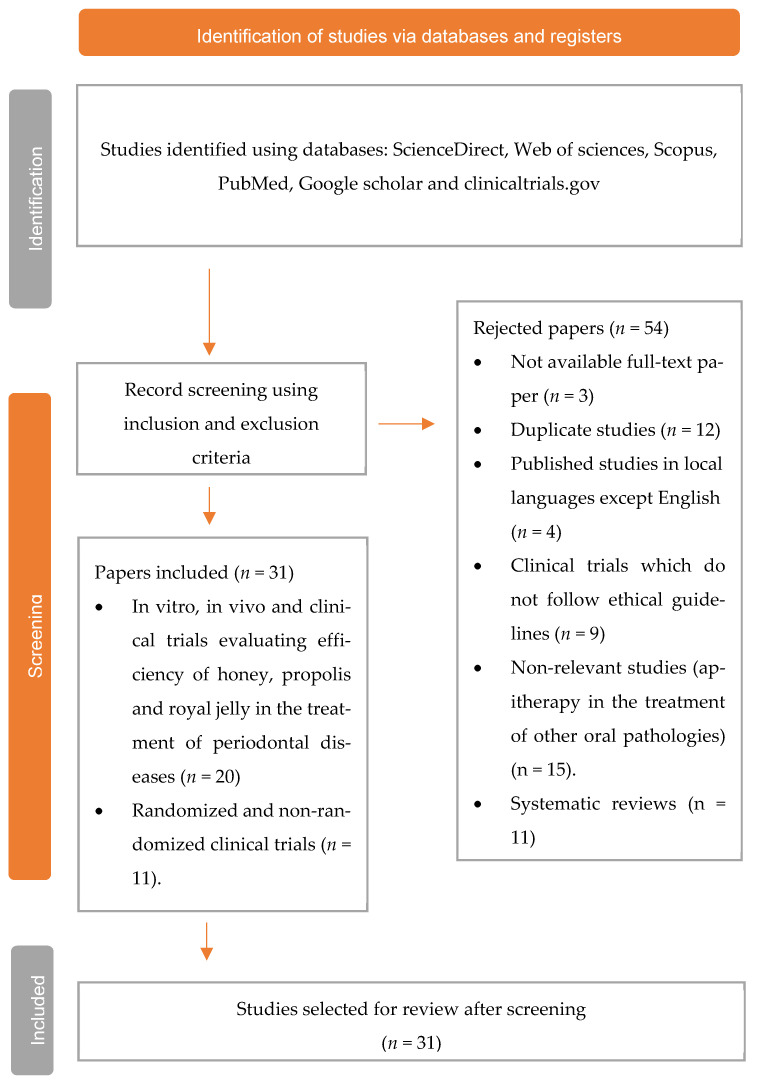
Flow diagram of the study selection process.

PRISMA flow diagram showing the selection process, including the identified records, inclusion and exclusion criteria, and the number of studies selected for review (Figure 1).

## 3. Results

### 3.1. Study Selection

A total of 85 studies were found from the database search; 12 duplicate studies were excluded, and 3 studies with no full text were removed. Thus, a total of 31 studies were selected for review. Bee products, such as honey, propolis, bee venom, and royal jelly, have numerous applications in the treatment of various diseases given their well-known bioactivities, such as antimicrobial, antioxidant, anticancer, and antiseptic activities. In the current study, we discussed 16 in vitro studies evaluating the role of apitherapy in the treatment of periodontal disease.

### 3.2. Scientific Studies Evaluating Honeybee Products in Periodontal Disease Treatment

#### 3.2.1. Antimicrobial Studies

Six of the studies investigated the antimicrobial effect of propolis against periodontal pathogenic bacteria in vitro. An ethanolic extract of propolis (EEP) shows an inhibitory effect on periodontal pathogenic bacteria, such as *Prevotella melaninogenica*, *Porphyromonas gingivalis*, *Porphyromonas asaccharolytica*, and *Prevotella intermedia*, based on a 30% *w*/*v* concentration. The zone of inhibition was 18.3 ± 0.64 mm for *P. melaninogenica*, 18.9 ± 0.05 mm for *P. gingivalis*, 22.8 ± 0.28 mm for *P. asaccharolytica*, and 22.8 ± 0.18 mm for *P. intermedia* [62]. Similarly, another study evaluated the antimicrobial activity of EEP and EEP-derived compounds using agar dilution assays and broth microdilution assays against *P. gingivalis* in vitro, and the results of both assays reported MIC values of 64 μg/mL (broth) and 128 μg/mL (agar). The mechanism of inhibition was also examined. EEP inhibited *P. gingivalis* activity and induced cell death within 30 min by increasing membrane permeability. EEP on the bacterial surface stimulated aberrant membrane bleb development following bleb fusion. Furthermore, the activity of EEP-derived compounds was examined. The results reported that ursolic acid inhibited bactericidal activity with membrane rupture. Baccharin and artepillin C show bacteriostatic activities with membrane blebbing [63].

The periodontopathic bacteria *Fusobacterium nucleatum*, *Eikenella corrodens*, and *Actinomyces odontolyticus* and oral carcinogenic bacteria (*Streptococcus mitis*, *Lactobacillus acidophilus*, *Streptococcus mutans*, and *Streptococcus sanguinis*) exhibited inhibitory effects in vitro when treated with propolis with a minimum inhibitory concentration of 12.5 μg/mL. In addition, propolis inhibits all periodontopathic bacteria and oral carcinogenic bacteria except for *L. acidophilus* with a MIC value of 6.3 μg/mL [64]. Similarly, in another study, propolis showed an inhibitory effect against *Streptococcus mutans* (bacteria) and *Candida albicans* (yeast), which are the causative organisms of dental caries. In addition, 50 μL propolis yields a 15.6 mm mean zone of inhibition for *Candida albicans* compared to 12 mm for probiotics and 14 mm for chlorhexidine. For *Streptococcus mutans*, the mean zone of inhibition was 9.4 mm for probiotics, 14 mm for chlorhexidine, and 14.6 mm for propolis. Compared to standard chlorhexidine and probiotics, propolis was found to be more effective in inhibiting *Streptococcus mutans* and *Candida albicans* [65].

The antibiofilm, cytotoxic and antimicrobial activities of propolis were assessed in an in vitro biofilm of *Fusobacterium nucleatum* and *Streptococcus gordonii*. Treatment with the methanolic fraction of propolis (chloroform partition) formed lower-than-average-thickness biofilms of *F. nucleatum* and *S. gordonii* at concentrations of 1.563 mg/mL (7.37 ± 1.620 µm and 9.24 ± 0.679 μm) and 0.78 mg/mL (6.84 ± 1.68 μm and 8.02 ± 1.6 μm), respectively. Cytoxicity assay of 0.78 mg/mL propolis (chloroform partition) on a human gingival fibroblast cell line (HGF-1) yielded 92.64% cell viability. An antimicrobial study of the methanolic fraction of propolis (chloroform residue) showed significant inhibition of *F. nucleatum and S. gordonii* bacteria with zones of inhibition of 12.15 ± 0.19 mm and 12.55 ± 0.19 mm, respectively, in comparison to propolis combined with chlorhexidine (14.33 ± 0.19 mm and 14.55 ± 0.19 mm, respectively) [66].

In another study, the antimicrobial activity of propolis against periodontal pathogens present in multispecies biofilms were examined in vitro. The subgingival biofilm with 32 species (7 days old) was treated with propolis from Day 3 (twice a day for 1 min). Results of microbial composition and metabolic activity determined by DNA–DNA hybridization of biofilms showed that 1600 μg/mL propolis showed no significant difference from the samples treated with chlorohexidine and decreased the metabolic activity by 45%. Based on results, propolis was found to be equally effective in decreasing subgingival biofilm formation compared to chlorhexidine [67]. Similarly, propolis at concentrations of 400, 800, and 1600 μg/mL was found to be effective in reducing the metabolic activity of multispecies biofilms (7 days old) by 57, 56, and 56%, respectively, compared to a 65% reduction with amoxicillin treatment. It was also observed that propolis treatment did not affect the host-compatible *Actinomyces* species level [68].

**Figure 2:**
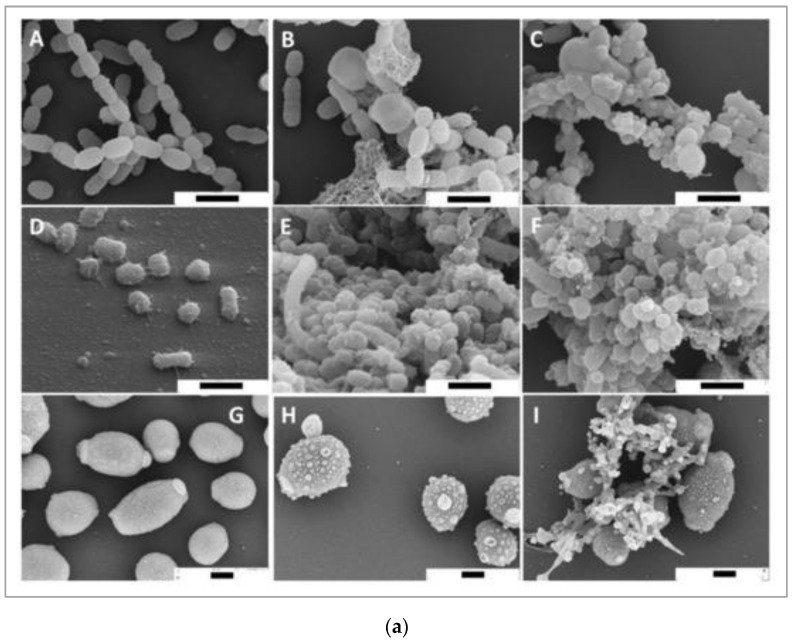

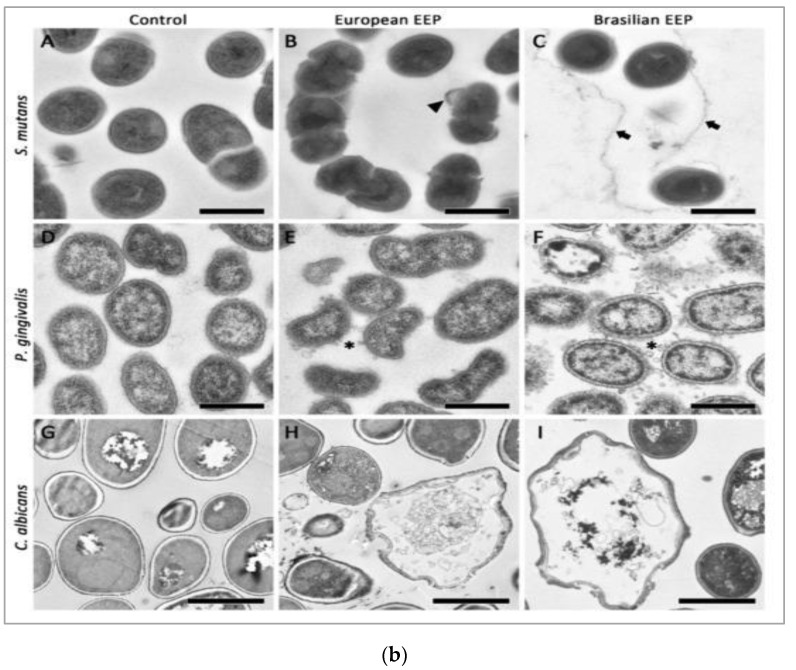
(**a**) SEM images of *S. mutans* (**A**–**C**), *P. gingivalis* (**D**–**F**), and *C. albicans* (**G**–**I**). Bar (**A**–**I**) 1 μm. European propolis treatment (5 min exposure, 25 mg/mL concentration) (**B**,**E**,**H**). Brazilian propolis treatment (5 min exposure, 25 mg/mL concentration) (**C**,**F**,**I**) [69]. (**b**) TEM images of *S. mutans* (**A**–**C**), *P. gingivalis* (**D**–**F**), and *C. albicans* (**G**–**I**). TEM images: (**A**,**D**,**G**) are without treatment. European propolis treatment (5 min exposure, 25 mg/mL concentration) (**B**,**E**,**H**) and Brazilian propolis treatment (5 min exposure, 25 mg/mL concentration) (**C**,**F**,**I**). Bar: 2 μm for *C. albicans*, (**A**–**I**) 500 nm for bacteria [69].

Bacteria causing periodontal diseases (*Porphyromonas gingivalis*), yeast causing candida infection (*Candida albicans*), and bacteria causing dental caries (*Streptococcus mutans*) treated with propolis showed an inhibitory effect with MIC values of 0.2, 6.25, and 0.2 mg/mL, respectively. The results of propolis treatment on three different biofilms of *P. gingivalis*, *C. albicans*, and *S. mutans* were also reported. Periodontal biofilm containing bacterial counts showed that 8.99 log10 colony forming units (CFU) of biofilm formation after 4 h was reduced to 3.21 log10 CFU by 100 mg/mL propolis after 4 h of treatment. The carcinogenic control biofilm containing 7.99 log10 CFU biofilm formation after 4 h was reduced to a bacterial count of 2.21 log10 CFU by 100 mg/mL propolis after 4 h of treatment. Candida biofilm containing bacterial counts 7.74 log10 CFU biofilm formation after 4 h was reduced to 3.65 log10 CFU by 100 mg/mL propolis after 4 h of treatment [69]. Scanning electron microscopy images suggest microbial cell wall interaction with propolis. After treatment with European propolis, large and small vesicles attached to the cell wall surface were observed. After Brazilian propolis treatment, damaged cells were found to stick together. Transmission electron microscopy images of *C. albicans* showed loss of cell wall integrity and cell enlargement after propolis treatment. Propolis treatment of *S. mutans* yielded minor modifications, and vesicles appeared outside of *P. gingivalis* cells. The results of scanning and transmission electron microscopy (SEM and TEM) showing the effect of propolis on bacteria are shown in Figure 2a,b.

The antifungal activity of propolis was evaluated against various Candida species extracted from chronic periodontitis in vitro. The results showed that the MIC values of propolis showed fungicidal and fungistatic activity against various Candida species: 64-152 and 32–64 µg/mL for *C. albicans*, 64 and 32–64 µg/mL for *C. tropicalis*, and 64–256 and 64–64 μg/mL for *C. glabrata*. Based on the results, it was observed that propolis shows antifungal activity against all three Candida species [70].

Comparing the in vitro studies discussed above, it was observed that 12.5–400 μg/mL propolis showed a significant antimicrobial effect against periodontal pathogenic bacteria. It has also been observed that EEP is more effective in the treatment of periodontopathic bacteria than raw propolis [63,64,68].

Four of the selected studies evaluated the effect of propolis on periodontitis treatment in vivo (rat model). The effect of propolis treatment on *P. gingivalis*-induced impaired glucose and lipid metabolism in C57BL/6 mice was studied. Powdered EEP with 2% carboxymethyl cellulose was administered to mice daily at a concentration of 200 mg/kg and effectively suppressed metabolic changes induced by *P. gingivalis*. Findings show that propolis treatment inhibited the upregulation of serum endotoxin levels and downregulated *P. gingivalis*-induced hepatic steatosis [71]. One of the studies reported that 5% and 10% propolis showed no significant therapeutic effect on periodontal disease in a *Mus musculus* model with ligature silk thread [72].

In another study, the administration of propolis (544 μg) and *Garcinia mangostana* L. (16 μg) complex (MEC) is effective in the prevention of alveolar bone loss and inhibition of inflammation in ligature-induced periodontal disease in a Wister rat model. The findings showed that MEC administration (ligation + lipopolysaccharide extracted from *P. gingivalis* + MEC 1:34 group) significantly reduced alveolar bone loss and downregulated the expression levels of COX-2, COX-1, MMP-8, iNOS, PGE2, and IL-8 [73]. In a similar study, propolis (10%) was effective in the treatment of ligature-induced periodontal disease in a Wister rat model. Propolis irrigation after scaling root planning caused downregulation in TNF-α, IL-1β, and malondialdehyde (MDA) serum levels compared to the control group with a statistically significant difference of *p* < 0.05 [74].

The antimicrobial effects of royal jelly have been examined in vitro against periodontopathic bacterial strains, including *Fusobacterium nucleatum*, *Prevotella intermedia*, *Porphyromonas gingivalis*, and *Aggregatibacter actinomycetemcomitans*. Specifically, 12.5–100 μg/mL royal jelly shows inhibitory effects on periodontopathic bacteria, and MIC values of royal jelly were higher for *F. nucleatum* and *A. actinomycetemcomitans* and lower for *P. intermedia* and *P. gingivalis* [75]. In another in vitro study, the antimicrobial activity of royal jelly was examined against periodontopathic bacteria in subgingival plaque. Royal jelly at concentrations of 12.5 and 25 μg/mL show inhibitory effects for anaerobic and aerobic bacteria compared to chlorohexidine, which showed inhibitory effects at concentrations of 6.25 and 3.25 μg/mL for anaerobic and aerobic bacteria, respectively [76]. Comparing the results of both studies, 12.5–100 μg/mL royal jelly showed an inhibitory effect on periodontopathic bacteria.

A recent in vitro study in 2021 evaluated the antibacterial efficiency of raw honey against patient-isolated *Escherichia coli* (reported as a periodontal pathogen due to its more effective lipopolysaccharide compared to *Porphyromonas gingivalis*). The zone of inhibition for 75% and 100% raw honey against patient-isolated *Escherichia coli* was 23 ± 0.666 and 27 ± 1.154 mm, respectively, which was equivalent to that of standard tetracycline. It has also been reported that raw honey and commercial honey at 100% concentration show a statistically significant difference (*p* < 0.01) in the zone of inhibition in treatment against *Escherichia coli* [77].

The main cause of periodontitis is the formation of bacterial biofilms due to poor oral hygiene [66]. Some periodontal pathogenic bacteria, such as *P. melaninogenica*, *P. gingivalis*, *P. asaccharolytica*, *P. intermedia*, *F. nucleatum*, and *S. gordonii*, were reported to form biofilms that cause periodontitis or gum infection [62,66]. Studies have reported that treatment with bee products, such as EEP, royal jelly, and raw honey, caused significant improvement in the reduction of periodontal biofilm formation by inhibiting different periodontopathic bacteria [67].

#### 3.2.2. Anti-Inflammatory Activity

**Figure 3:**
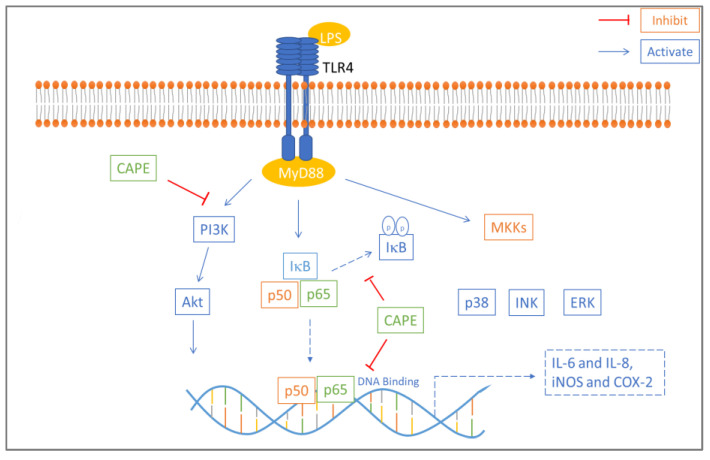
Mechanism of anti-inflammatory effect of CAPE in LPS-induced HGFs. CAPE inhibited phosphorylation of IκB which reduced NF-κB p50 and p65 nuclear translocation. Akt and PI3K phosphorylation involved in NF-κB activation is also inhibited by CAPE. NF-κB p65 DNA binding is also blocked by CAPE [54].

An in vitro study examined the anti-inflammatory effect of caffeic acid phenethyl ester (CAPE) on lipopolysaccharide-induced human gingival fibroblasts (cells present in periodontal soft tissue). CAPE is one of the main active compounds found in propolis and has various well-known bioactivities, such as immune regulation and antioxidant, anti-inflammatory, and antitumor activities. CAPE inhibited LPS-induced inducible nitric oxide synthase (iNOS), cyclooxygenase 2 (COX-2), and interleukin (IL-8 and IL-6) production in a dose-dependent manner and inhibited protein kinase B (AKT) and phosphatidylinositol 3 kinase (PI3K) phosphorylation. Western blot assay results showed that lipopolysaccharide-stimulated nuclear factor kappa B (NF-κB) and TLR4/MyD88 activation were suppressed by CAPE treatment (Figure 3). Based on these results, CAPE reduces the proinflammatory response in lipopolysaccharide-induced human gingival fibroblasts via the NF-κB and PI3K/Akt signaling pathways [78].

CAPE inhibits phosphorylation of IκB, which reduces NF-κB p50 and p65 nuclear translocation. Akt and PI3K phosphorylation is important for NF-κB activation and is also inhibited by CAPE. NF-κB p65 DNA binding is also blocked by CAPE [54].

Purified bee venom was examined in vitro to determine whether it can reduce inflammatory periodontitis induced by *P. gingivalis* and osteoclast differentiation induced by receptor activator of nuclear factor-kappa B ligand signaling (RANKL). The results showed that bee venom (100 μg/kg) treatment reduced inflammatory bone loss-related periodontitis induced by *P. gingivalis* and reduced the expression of IL-1β and tumor necrosis factor (TNF)-α in vivo. Bee venom treatment also suppressed osteoclast-specific gene expression of tartrate resistant acid phosphate (TRAP), cathepsin K, integrin αVβ3, and nuclear factor of activated T cells 1 (NFATc1) and suppressed multinucleated osteoclast differentiation induced by RANKL [79]. Similarly, another in vitro study examined the anti-inflammatory mechanism of purified bee venom treatment on a *P. gingivalis* lipopolysaccharide (PGLPS)-induced human keratinocyte cell line (HaCaT). The results showed that PGLPS upregulated the expression of proinflammatory cytokines, including IL-1β, IL-8, IL-6, TNF-α, and toll-like receptor (TLR)-4, and induced signaling pathway activation of the inflammatory cytokine-related transcription factors activator protein 1 (AP-1) and NF-κB. Furthermore, treatment with bee venom (100 ng/mL) inhibited proinflammatory cytokines by downregulating the AP-1 and NF-κB signaling pathways [80].

Melittin, a compound found in bee venom and known for its antibacterial and anti-inflammatory effects, was investigated in vitro for its anti-inflammatory effect on PGLPS-treated HaCaT cells. PGLPS treatment of HaCaT cells upregulated the expression of proinflammatory cytokines, such as interferon (IFN)-γ, IL-6, IL-8, TNF-α, and TLR-4, and induced NF-κB, protein kinase B/Akt, and extracellular signal-regulated kinase (ERK) signaling pathway activation. However, treatment with 1 µg/mL melittin downregulated the expression of proinflammatory cytokines by suppressing the signaling pathway activation of NF-κB, Akt, and ERK. Based on these results, melittin treatment reduced the PGLPS-induced inflammatory response [81].

Cytokines act as the first wave of response to periodontopathic bacteria, are modulators of the inflammatory response and homeostasis and stimulate accessory cell populations and lymphocytes. The disordered regulation of cytokines can induce or accelerate periodontitis, as some studies have shown that single nucleotide polymorphisms in cytokines are related to the severity of periodontal disease [10]. Some studies have reported that bee products, such as propolis and bee venom as well as their compounds CAPE and melittin, can provide a balance to disrupt the regulation of cytokines through the downregulation of their expression and subsequent suppression of signaling pathways. These effects collectively reduce the severity of periodontitis [78,79,80,81].

The outcomes of the various in vitro studies discussed above are summarized in Table 1.

### 3.3. Safety of Honeybee Products in Periodontal Disease Treatment

In the current study, we discussed 11 clinical studies evaluating the role of apitherapy in the treatment of periodontal disease. A randomized double-blind controlled clinical trial investigated the effect of propolis (topical administration) into >5 mm periodontal pockets of periodontitis patients. A total of 24 patients diagnosed with chronic periodontitis were selected and divided into four groups (6 patients in each group). Each group underwent treatment with a different ointment: Group I—placebo group (placebo carboxymethyl cellulose sodium salt (CMC) ointment); group II—propolis group (0.01 mg/mL EEP in CMC ointment); group III—curry leaf group (1 mg/mL water-extracted curry leaf in CMC ointment); and group IV—minocycline group (2% minocycline hydrochloride ointment). Propolis ointment was administered thrice at 1-month intervals. The results showed that *P. gingivalis* was significantly reduced in gingival crevicular fluid after treatment with propolis. An improvement in the score of the clinical attachment level was noted in the propolis group (1.67 ± 1.22 mm) compared to the placebo group (0.33 ± 0.82 mm), but the difference was not significant (mean difference (MD)—1.33 mm, *p* = 0.160 and confidence interval (CI) 95% of difference—0.42 to 3.08 mm). Propolis also improved the score of probing pocket depth (1.83 ± 1.17 mm) compared to the placebo (0.33 ± 0.82 mm), but the difference was not significant (CI 95% of difference—0.11 to 2.89 mm, *p* = 0.033 and MD—1.50 mm) [82].

The efficacy of propolis-containing mouthwash in the treatment of gingivitis was evaluated in a double-blinded randomized clinical trial (Registered in Iranian Randomized Clinical Trial site with IRCT ID: IRCT20150210021029N3). A total of 32 patients diagnosed with gingivitis were selected and divided into two groups: Group I received propolis extract containing mouthwash, and Group II received the same mouthwash without propolis extract (each group had 16 patients allocated). The propolis mouthwash (30 drops mixed with 20 mL water) was given to patients twice a day (gargle 1 min) with a 12-hour interval. The results showed no significant difference (*p* = 0.91) in the plaque index (PI) score of the propolis group (85.19 ± 51.6%) compared to the placebo group (83.93 ± 36.1%). The results showed a significant reduction in the papillary bleeding index (PBI) of the propolis compared with the placebo group with a significant difference of *p* < 0.001 between the two groups. The tooth color change over time was insignificant in the propolis group (*p* = 0.14) and significant in the placebo group [83].

In another double-blind randomized clinical trial, propolis extract, nano vitamin E, and nano vitamin C in gel formulation were examined for their efficiency as adjuvants to mechanical debridement in peri-implant mucositis (PM) treatment. In this study, a total of 46 patients with at least one implant with PM were selected and were divided into two groups: Group I was treated with a 2% propolis extract-containing gel, and Group II served as the control group without propolis gel. The test group and control group included 23 participants each and were advised to use gel as toothpaste for 1 month 3 times/day. The results showed that after treatment, 0% of patients in the control group and 26.1% of patients in the test group showed complete PM resolution (*p* = 0.02). In the test group, a significant reduction was reported in probing depths (*p* = 0.27), plaque index score (*p* = 0.03), and bleeding on probing (*p* = 0.04) compared to the control groups. From baseline to the 1-month follow-up, significant reductions in *Porphyromonas gingivalis* (*p* = 0.05) and *Tannerella forsythia* (*p* = 0.02) were observed in the test group compared with the control group. Based on the results, the test gel shows antimicrobial activity after the course of 1 month and clinically improved PM [84].

The effectiveness of manuka honey and raw honey mouthwash on GI and PI was evaluated in a double-blind randomized controlled field trial (CTRI no: CTRI/2017/11/010565). A total of 135 school children were selected for the study and were divided into three groups with 45 participants each: Group I used manuka honey, Group II used raw honey, and Group III used chlorhexidine mouthwash (control). Participants were instructed to use 10 mL of honey mouthwash twice/day for the course of 21 days. Examination of participants was performed at baseline, one day after mouthwash discontinuation, and 1week after mouthwash discontinuation. The results of the clinical parameters PI and GI score showed statistically significant reductions in the test groups (manuka and raw honey mouthwash) and control group (chlorhexidine mouthwash). The GI score in the raw honey mouthwash group decreased from baseline (1.465 ± 0.17) to the 22nd day (0.927 ± 0.26). The GI score in the manuka honey mouthwash group decreased from baseline (1.457 ± 0.18) to the 22nd day (0.976 ± 0.15). The score of the chlorhexidine mouthwash group decreased from baseline (1.452 ± 0.19) to the 22nd day (0.498 ± 0.5). The PI score of the raw honey mouthwash group decreased from baseline (1.525 ± 0.2) to the 22nd day (0.723 ± 0.11). The score of the manuka honey mouthwash group decreased from baseline (1.525 ± 0.2) to the 22nd day (0.72 ± 0.12), and the score of the chlorhexidine mouthwash group decreased from baseline (1.505 ± 0.23) to the 22nd day (0.495 ± 0.13). Based on the results, honey-based mouthwash shows similar antimicrobial effects on PI and GI scores compared to chlorhexidine mouthwash [85].

A randomized controlled clinical trial investigated the immunological and clinical efficacy of propolis and mangosteen extract (PME) on gingivitis and early periodontitis. A total of 80 patients diagnosed with incipient periodontitis or gingivitis were selected and randomly allocated to two groups, including Group I—test (capsule with PME) and Group II—control (same capsule without PME) with 41 and 39 participants, respectively. Test group patients were advised to take 194 mg of PME capsules, and control group patients were advised to take the same capsule without PME daily for the course of 8 weeks. The results showed a significant difference of *p* = 0.0406 in the modified GI between the test and control groups at 4 and 8 weeks. The results of the test group also showed an increase in salivary matrix metalloproteinase-9 and a reduction in IL-6 after 8 weeks. Patient-reported outcomes assessed by oral health impact profile (OHIP)-14 questionnaires also showed improvement after 4 weeks in the test group compared to the placebo group [86].

In another randomized clinical trial, the antimicrobial effect of propolis (mouthwash and pate formulation) was investigated in patients (after tooth extraction) with peri-odontal disease. A total of 60 patients for the study of propolis paste and 40 patients for the propolis mouthwash study were selected. Furthermore, the mouthwash patients were divided into four groups: Group I—placebo (control mouthwash); Group II—used 0.2% chlorhexidine containing mouthwash; Group III—used 2% propolis containing mouthwash; and Group IV—used 0.2% chlorhexidine + 2% propolis-containing mouthwash. Each group had 10 participants, separately. The result of the propolis mouthwash assay shows a reduction in bacterial proliferation. In particular, patients using the mouthwash formulation of 0.2% chlorhexidine + 2% propolis exhibited < 10^5^ CFU. The results of the propolis paste assay reported 90% complete healing in periodontal sockets in comparison with the control paste, which showed 13.4% complete healing after three days of surgery. Based on these results, propolis paste was found to be a viable alternative for periodontal socket healing after dental extraction [87].

The anti-inflammatory effect of polyherbal mouthwash containing *Salvia officinalis*, *Plantago lanceolata* leaf extract, 1.75% essential oil, and propolis extract was evaluated in a single-blind randomized controlled trial. A total of 40 patients were selected with moderate or severe periodontitis and were divided into two groups: Group I—phytoherbal mouthwash; and Group II—placebo mouthwash. Twenty participants were allocated to each group. The test group was instructed to rinse with phytoherbal mouthwash, and the control group was instructed to rinse with placebo mouthwash for 2 min twice/day for the course of 3 months. The results of probing depth (PD), clinical attachment level (CAL), full month plaque score (FMBS), and full month bleeding score (FMBS) were recorded at baseline and after the course of 3 months. Both the control group and test group showed a statistically significant reduction from baseline to 3 months in the P.D (CG *p* = 0.011, TG *p* = 0.001), FMPS (CG *p* = 0.003, TG *p* = 0.001), CAL (CG *p* = 0.020, TG *p* < 0.001), and FMBS (CG *p* = 0.002, TG *p* = 0.001) [88].

In another randomized controlled clinical trial, the efficiency of propolis and herbs (antioxidant-based formula) as adjunctive therapy to nonstandard periodontal treatment was examined. In this study, a total of 40 patients were selected and randomly allocated to the test group or control group. The results of clinical parameters were recorded at baseline, 1 month, and after 3 months. No significant clinical difference was noted between the two groups (*p* > 0.05). It has also been reported that the results of the test group show better oxidation stress reduction results than those of the placebo group [89].

In a triple-blind randomized controlled clinical trial, the efficacy of a propolis mouth rinse on oral pathogens was investigated. A total of 120 participants were selected and randomly assigned to four different groups: Group I—hot EEP; Group II—cold EEP; Group III—0.2% chlorhexidine gluconate; and Group IV—placebo (distilled water). In total, 30 participants were included in each group. Participants were advised to rinse twice a day for the course of 3 months. For the microbial assay, saliva was collected at baseline, 5 min, and 1 h, and GI and PI were recorded at baseline, 15 days, 1 month, and 3 months. The results show a decline in the *S. mutans* concentration after the use of mouth rinse (*p* < 0.05). The cell counts of *S. mutans* and *L. acidophilus* were decreased compared with baseline with the use of chlorhexidine mouthwash (5.8 × 10^2^) and hot ethanolic propolis mouthwash (5.5 × 10^2^). Additionally, a significant reduction in plaque scores was observed after the course of 3 months in the cold ethanolic propolis (0.46), hot ethanolic propolis (0.47), and chlorhexidine (0.45) mouthwash groups. Based on the results, propolis mouthwash was found to be as effective as chlorhexidine mouthwash in reducing dental caries pathogens and dental plaque [90].

In a randomized placebo–control study, the effect of Polish propolis and plant oils (toothpaste) on the oral cavity health of patients with orthodontically treated oral clefts was examined. A total of 50 patients were selected and were randomly assigned into two groups: Group I—test group (used toothpaste with active ingredients, including menthol, rosemary oil, Polish propolis, and tea tree oil); and Group II—control group (used toothpaste without active ingredients as placebo). In total, 25 patients were allocated to each group. Patients were advised to brush their teeth with propolis toothpaste or placebo toothpaste 3 times/day for 3 min over the course of 35 days. The results show that after the use of propolis toothpaste in Group I (toothpaste with propolis and plant oils) for gingival conditions, the gingival bleeding index (GBI) was significantly decreased for molars (*p* = 0.0017), incisors (*p* = 0.007), and total GBI (*p* = 0.002). A significant improvement in the oral hygiene index (OHI) was observed (*p* = 0.011). Based on the results, propolis and plant oil toothpaste can be effective in preventing and controlling oral infectious diseases that occur during orthodontic treatment of oral clefts [91].

In a triple-blind parallel-group clinical trial, the effect of propolis mouthwash treatment on GI and PI was evaluated in patients undergoing orthodontic treatment. In this study, a total of 40 patients were selected and randomly assigned to two groups: Group I—test group (propolis aqueous extract); and Group II—control group (chlorhexidine mouthwash). Twenty patients were allocated to each group. The test group and control group were advised to use mouthwash for 3 weeks after brushing their teeth twice/day consecutively. The GI, PI, and periodontal index results were evaluated at baseline and after 3 weeks. A statistically significant difference between the scores of periodontal index (*p* = 0.005), PI (*p* < 0.001), and GI (*p* = 0.006) in the test group was observed. In the chlorhexidine group, significant differences were also observed in the periodontal index (*p* = 0.003), GI (*p* = 0.001), and PI (*p* < 0.001). Based on results, propolis mouthwash was found to be as effective as chlorhexidine mouthwash [92].

Out of the 11 selected clinical trials discussed above, 8 were randomized clinical trials, including 4 single randomized clinical trials, 3 double-blinded randomized clinical trials, 1 triple-blinded clinical trial, and 3 non-randomized clinical trials. The majority of clinical trials investigated the effect of propolis, and one of the clinical trials investigated the effect of raw honey in periodontitis treatment [85]. Out of the four double-blind randomized clinical trials, one study showed no significant difference in clinical attachment level, CI or probing pocket depth with treatment with propolis ointment compared to the control group [82]. All the clinical trials suggested that propolis and honey-based products, such as mouthwash [83,85,87,88,90,92], gel [89], ointment [82], capsule [86], and toothpaste [84,91], were significantly effective compared to control groups in the treatment of periodontal disease.

The outcomes of clinical trials investigating the safety of honeybee products in periodontal disease treatment are shown in Table 2.

The observed limitation of this study is the use of various indices and assessment criteria to determine the effect of bee products on periodontal disease. The plaque index was measured in only three studies [59,64,68] with an 85% reduction compared to the control group, 83%. One study measured the plaque score [66], and the other seven studies did not use the plaque index. The gingival index was measured in only two studies [61,68]. Bleeding was measured using two different criteria: probing bleeding [64] and papillary bleeding index [59]. Probing depth was measured in three studies [58,64,66]. Regarding other assessment criteria, CAL was measured in two studies [58,66] with a confidence interval reported in one study [58]. A reduction in bacterial proliferation and healing of the periodontal socket was noted with 90% recovery compared to the control showing 13.4% recovery [63]. Furthermore, there are limitations in sample size and assessment time. Different numbers of participants were selected in all studies. Most studies had small sample sizes, and heterogeneity was noted in the assessment time of each clinical trial, ranging from 1 week to a few months. Bee products, including bee venom, royal jelly, and honey, have shown good results in the treatment of periodontal disease; however, limited clinical studies and experimental evidence are available to date supporting the effect of bee products. However, in the case of propolis, the antimicrobial and antioxidant activities are well known, but a limited number of randomized clinical trials are available. Therefore, in the future, it is important to perform more randomized clinical trials to assess periodontal parameters, such as bleeding, gingival index, plaque, and oral hygiene index, using unified criteria, performing well-defined research with a broader sample size, and following standard ethical guidelines to compare the use of bee products with the control group.

## 4. Conclusions

Over a long period of time, apitherapy has maintained its popularity, and various bee products, such as propolis, bee venom, and honey, have been scientifically demonstrated to have numerous applications in dentistry due to their antimicrobial, anti-inflammatory, anticancer, immune-modulating, and antioxidant properties. Based on clinical and experimental evidence, it is suggested that propolis is the most effective bee product in the treatment of periodontal disease with the concentration range of 12–400 μg/mL. These bee products are likely to represent an alternative to synthetic drugs in periodontal disease treatment in the future; however, to date, limited in vivo and clinical evidence validating the application of bee products, especially bee venom, honey, and royal jelly is available. Furthermore, numerous findings supported by in vitro, in vivo, and clinical trials have validated that propolis products, such as mouthwash, gels, ointments, and toothpaste, have potential application in periodontitis treatment, showing antioxidant and antimicrobial activity. Based on these studies, it can be concluded that bee products are safer to use in the treatment of periodontitis; however, there is a need for more clinical and in vivo experimental evidence to explore the underlying mechanism of the bioactivities of bee products in periodontitis treatment.

## Figures and Tables

**Table 1:** Scientific studies have examined the effects of honeybee products in the treatment of periodontal disease.

Bee Products/Country of Origin	Bacterial Strain/Yeast Strain/Cell Line	Obtained Results	Reference
Antimicrobial studies			
Propolis (Margalla hills, Islamabad)	*Prevotella melaninogenica*, *Porphyromonas gingivalis*, *Porphyromonas asaccharolytica* and *Prevotella intermedia*	EEP (30% *w*/*v* concentration) shows an inhibitory effect on periodontal bacteria with zone of inhibition 18.3 ± 0.64 mm for *P. melaninogenica*, 18.9 ± 0.05 mm for *P. gingivalis*, 22.8 ± 0.28 mm for *P. asaccharolytica*, and 22.8 ± 0.18 mm for *P*. *intermedia*.	[62]
Propolis (Minas Gerais State, Brazil)	*Porphyromonas gingivalis*	Result of both assays reported the MIC value of 64 μg/mL (broth) and 128 μg/mL (agar).	[63]
		EEP inhibited *P. gingivalis* activity and induced cell death within 30 min by increasing membrane permeability.	
		Ursolic acid inhibited bactericidal activity with membrane rupture. Baccharin and artepillin C show bacteriostatic activities with membrane blebbing.	
Propolis (Kopaonik, Serbia)	Periodontopathic bacteria: *Fusobacterium nucleatum*, *Eikenella corrodens* and *Actinomyces odontolyticus* and oral carcinogenic bacteria: *Streptococcus mitis*, *Lactobacillus acidophilus*, *Streptococcus mutans and Streptococcus sanguis*	Propolis with MIC value of 12.5 μg/mL inhibits all periodontopathic bacteria and oral carcinogenic bacteria except *L. acidophilus* with a MIC value of 6.3 μg/mL.	[64]
Propolis (Bangalore, India)	*Streptococcus mutans* (bacterial strain) and *Candida albicans* (yeast strain)	Propolis with a concentration of 50 μl shows 15.6 mm mean zone of inhibition for *Candida albicans* as compared to probiotics 12 mm and chlorhexidine 14 mm.	[65]
		For *Streptococcus mutans*, mean zone of inhibition was 9.4 mm for probiotics, 14 mm for chlorhexidine, and 14.6 mm for propolis.	
Propolis (Andean regions, Peru)	*Fusobacterium nucleatum* and *Streptococcus gordonii*	Treatment of methanolic fraction of propolis (chloroform partition) formed lower than average thickness biofilms of *F. nucleatum* and *S. gordonii* with concentrations of 1.563 mg/mL (7.37 ± 1.620 μm and 9.24 ± 0.679 μm) and 0.78 mg/mL (6.84 ± 1.68 µm and 8.02 ± 1.6 μm).	[66]
		Cytotoxic assay of propolis (chloroform partition) on human gingival fibroblast cell line (HGF-1) at the 0.78 mg/mL dilution shows cell viability of 92.64%.	
		Antimicrobial study of methanolic fraction of propolis (chloroform residue) shows significant inhibition of *F. nucleatum* and *S. gordonii* bacteria with zone of inhibition, 12.15 ± 0.19 mm and 12.55 ± 0.19 mm in comparison to propolis combined with chlorhexidine (14.33 ± 0.19 mm and 14.55 ± 0.19 mm).	
Propolis (City of Maceio, Alagoas State, north-eastern Brazil)	Periodontal pathogens present in multispecies biofilm	Propolis with a concentration of 1600 μg/mL shows no significant difference to sample treated with chlorohexidine and decreased the metabolic activity by 45%.	[67]
Propolis (City of Maceio, Alagoas State, north-eastern Brazil)	Periodontal pathogens present in multispecies biofilm	Propolis with a concentration of (400, 800, and 1600 μg/mL) was found to be effective in reducing metabolic activity of multispecies biofilms (7 days old) by 57, 56, and 56%, respectively, in comparison to 65% reduction with treatment of amoxicillin.	[68]
Propolis (South America)	Bacteria causing periodontal diseases (*Porphyromonas gingivalis*), yeast causing candida infections (*Candida albicans*), and bacteria causing dental caries (*Streptococcus mutans*)	MIC value of European EEP reported for *P. gingivalis* was 0.2 mg/mL, for *C. albicans* was 6.25 mg/mL, and for *S. mutans* was 0.2 mg/mL.	[69]
		Periodontal biofilm containing bacterial counts 8.99 log10 CFU biofilm formation after 4 h was reduced to 3.21 log10 CFU by propolis with concentration of 100 mg/mL after 4 h treatment.	
		Carcinogenic control biofilm containing 7.99 log10 CFU biofilm formation after 4 h was reduced to bacterial count of 2.21 log10 CFU by propolis with concentration of 100 mg/mL after 4 h treatment.	
		Candida biofilm containing bacterial counts 7.74 log10 CFU biofilm formation after 4 h was reduced to 3.65 log10 CFU by propolis with concentration of 100 mg/mL after 4 h treatment.	
Propolis (Belo Horizonte, Brazil)	Yeast strain—*Candida albicans*, *Candida tropicalis*, *Candida glabrata*	Propolis shows fungicidal and fungistatic activity on various Candida species, respectively, for *C. albicans* MIC values were 64–152 and 32–64 μg/mL, for *C. tropicalis* were 64 and 32–64 μg/mL, and for *C. glabrata* were 64–256 and 64 μg/mL.	[70]
Propolis (Okayama, Japan)	*P. gingivalis* W83 and C57BL/6 mice	Propolis treatment inhibited upregulation of serum endotoxin levels and downregulated *P. gingivalis* induced hepatic steatosis.	[71]
Propolis (Gwangju, Republic of Korea)	*P. gingivalis* KCOM 2804 and Wistar rats (weighing 250–400 g)	Finding shows MEC administration (L + LPS from *P. gingivalis* + MEC 1:34 group) showed significant reduction in alveolar bone loss and downregulated the expression levels of COX-2, COX-1, MMP- 8, iNOS, PGE2, and IL-8.	[73]
Propolis (Haj Umran city, Iraq)	Wistar rats (weighing 250–300 g)	Propolis irrigation after scaling root planning shows downregulation in TNF-α, IL-1β, and MDA serum levels as compared to control group with statistically significant difference of *p* < 0.05	[74]
Royal jelly (RHF, Singapore.)	*Fusobacterium nucleatum*, *Prevotella intermedia*, *Porphyromonas gingivalis*, and *Aggregatibacter actinomycetemcomitans*	Royal jelly with concentration range of 12.5–100 μg/mL shows inhibitory effects on periodontopathic bacteria.	[75]
Royal jelly (Uttar Pradesh, India)	Periodontopathic bacteria in subgingival plaque	Royal jelly with higher concentrations of 12.5 and 25 μg/mL shows inhibitory effects for anaerobic and aerobic periodontopathic bacteria.	[76]
Raw honey (Kanpur, India)	*Escherichia coli*	Zone of inhibition (ZI) for raw honey against patient isolated *Escherichia coli* with concentration of 75% and 100% is found to be 23 ± 0.666 and 27 ± 1.154 mm which was equivalent to standard tetracycline.	[77]
Anti-inflammatory activity			
Caffeic acid phenethyl ester (Saint Louis, MO, USA)	*P. gingivalis* and human gingival fibroblasts	CAPE in a dose-dependent manner inhibits LPS induced inducible nitric oxide synthase (iNOS), cyclooxygenase 2 (COX-2), interleukins (IL-8 and IL-6) production and inhibits protein kinase B (AKT) and phosphatidylinositol 3 kinase (PI3K) phosphorylation.	[78]
		Result of Western blot assay shows that lipopolysaccharide stimulated nuclear factor kappa B (NF-kB) and TLR4/MyD88 activation was suppressed by CAPE treatment.	
Purified bee venom (Suwon, Korea)	*P. gingivalis*, Balb/C mice and Mouse monocyte/macrophage RAW 264.7 cells	Purified bee venom (100 μg/kg) treatment reduces inflammatory bone loss related periodontitis by *P. gingivalis* and reduced expression of IL-1β and TNF-α in vivo.	[79]
		Purified bee venom treatment suppressed osteoclast specific gene expression of TRAP, cathepsin K, integrin αVβ3, and NFATc1 and suppressed multinucleated osteoclast differentiation induced by RANKL.	
Purified bee venom (Suwon, Korea)	*P. gingivalis* and HaCaT cell line	PGLPS upregulate expression of pro-inflammatory cytokines including IL-1β, IL-8, IL-6, TNF-α, and TLR-4, in addition induced signaling pathway activation of inflammatory cytokines related transcription factors, AP-1, and NF-kB.	[80]
		Further the treatment of bee venom (100 ng/mL) inhibited the pro-inflammatory cytokines by downregulation of AP-1 and NF-kB signaling pathways.	
Melittin (Farmingdale, NY, USA)	*P. gingivalis* and HaCaT cell line	Melittin treatment with concentration of 1 μg/mL downregulated the expression of pro-inflammatory cytokine by suppressing signaling pathway activation of NF-kB, Akt, and ERK.	[81]

EEP—ethanolic extract of propolis; ZI—zone of inhibition; MIC—minimum inhibitory concentration; EEP—ethanol extract of propolis; HaCaT—human keratinocyte cell line; RHF—Royal health foods.

**Table 2:** Clinical trials were conducted to evaluate the potential of bee products in periodontal disease treatment.

Bee Product/Country of Study	Participants	Interventions	Outcome	Reference
Propolis (Matsudo, Japan)	Total participants (*n* = 24) Four groups: Group I—placebo (*n* = 6) Group II—propolis (*n* = 6) Group III—curry leaf (*n* = 6) Group IV—minocycline (*n* = 6)	Propolis ointment was given three times with a 1 month interval to tooth having periodontal pocket ≥ 5mm.	With propolis treatment *P. gingivalis* is significantly reduced in gingival crevicular fluid and improvement in score of clinical attachment level in propolis (1.67 ± 1.22 mm) is observed.	[82]
Propolis (Isfahan, Iran)	Total participants (*n* = 32) Two groups: Group I—propolis (*n* = 16) Group II—control (*n* = 16)	The propolis mouthwash (30 drops mixed with 20 mL water) was given to patients twice a day (gargle 1 min) with a 12-hour interval.	Results shows that there is no significant difference (*p* = 0.91) in plaque index (PI) score of propolis (85.19 ± 51.6%) in comparison to placebo group (83.93 ± 36.1%).	[83]
			Result of papillary bleeding index (PBI) shows significant reduction in PBI of propolis group in comparison with placebo group with significant difference of *p* < 0.001 between two groups.	
Propolis (South-East, South Korea)	Patients were selected with at least one implant with PM. Total participants (*n* = 46) Two groups: Group I—propolis test group (*n* = 23) Group II—control test group (*n* = 23)	The test group were advised to use gel as toothpaste for 1 month 3 times/day.	In the test group a significant reduction is reported in probing depths (*p* = 0.27), plaque index score (*p* = 0.03), and bleeding on probing (*p* = 0.04) compared to control groups.	[84]
			From baseline to 1 month follow up significant statistical reduction in *Porphyromonas gingivalis* (*p* = 0.05) and *Tannerella forsythia* (*p* = 0.02) was observed in the test group in comparison with the control group.	
Honey (Belagavi, Karnataka)	Total participants (*n* = 135) Three groups: Group I—manuka honey (*n* = 45) Group II—raw honey (*n* = 45) Group III—control (chlorhexidine) (*n* = 45)	Instructed to use 10 mL of honey mouthwash twice/day for the course of 21 days.	The GI score of raw honey mouthwash reduced from baseline 1.465 ± 0.17 to 22nd day 0.927 ± 0.26, score of manuka honey mouthwash reduced from baseline 1.457 ± 0.18 to 22nd day 0.976 ± 0.15.	[85]
			The PI score of raw honey mouthwash reduced from baseline 1.525 ± 0.2 to 22nd day 0.723 ± 0.11, score of manuka honey mouthwash reduced from baseline 1.525 ± 0.2 to 22nd day 0.72 ± 0.12.	
Propolis (Seoul, South Korea)	Total participants (*n* = 80) Two groups: Group I—PME (*n* = 41) Group II—control or placebo (*n* = 39)	Patients diagnosed with incipient periodontitis or gingivitis was selected and the patients were advised to take 194 mg of PME capsule daily for the course of 8 weeks.	Result shows significant difference of *p* = 0.0406 in modified GI between test and control groups during 4 and 8 weeks.	[86]
			Results of test group also reported that increase in salivary matrix metalloproteinase-9 and reduction in IL-6 was observed after 8 weeks.	
Propolis (Granada, Spain)	Total participants (*n* = 40) Four groups: Group I—placebo or control mouthwash (*n* = 10), Group II—0.2% chlorhexidine containing mouthwash (*n* = 10), Group III—2% propolis containing mouthwash (*n* = 10) and Group IV—0.2% chlorhexidine + 2% propolis (*n* = 10)	Patients for propolis mouthwash study was advised to use mouthwash 3 times/day for 2 days.	Result of propolis mouthwash assay shows reduction in bacterial proliferation, especially the mouthwash formulation of 0.2% chlorhexidine + 2% propolis reported < 105 CFU.	[87]
			Result of propolis paste assay reported 90% of complete healing in periodontal sockets in comparison with control paste which shows 13.4% complete healing after 3 days of surgery.	
Propolis (Milan, Italy)	Total participants (*n* = 40) Two groups: Group I—test (phytoherbal group) (*n* = 20) Group II—control (placebo mouthwash) (*n* = 20)	Test group was instructed to rinse with mouthwash for 2 min, twice/day for the course of 3 months.	Both control group and test group show a statistically significant reduction from baseline to 3 months in the score of P.D. (CG *p* = 0.011, TG *p* = 0.001), FMPS (CG *p* = 0.003, TG *p* = 0.001), CAL (CG *p* = 0.020, TG *p* < 0.001), and FMBS (CG *p* = 0.002, TG *p* = 0.001).	[88]
Propolis (Pisa, Italy)	Total participants (*n* = 40) Two groups: Group I—control group (chlorhexidine gel formula + NSPT) Group II—test group (antioxidant gel formula + NSPT)	Propolis and herbs (antioxidant gel) as adjunctive therapy to non-standard periodontal treatment (NSPT).	Test group show better oxidation stress reduction results as compared to placebo group.	[89]
Propolis (Udaipur, India)	Total participants (*n* = 120) Four groups: Group I—hot EEP (*n* = 30), Group II—cold EEP (*n* = 30), Group III—0.2% chlorhexidine gluconate (*n* = 30) and Group IV—placebo (distilled water) (*n* = 30)	Advised to use mouthrinse twice a day for the course of 3 months.	Result shows decline in *S. mutans* concentration after use of mouth rinse *p* < 0.05.	[90]
			The cell count of *S. mutans* and *L. acidophilus* is found to be decreased in comparison to baseline with use of chlorhexidine mouthwash (5.8 × 102) and hot ethanolic propolis mouthwash (5.5 × 102).	
			Significant reduction in plaque scores was observed after the course of 3 months in cold ethanolic propolis (0.46), hot ethanolic propolis (0.47), and chlorhexidine (0.45) mouthwash groups.	
Propolis (Katowice, Poland)	Total participants (*n* = 50) Two groups: Group I—test group (active ingredient) (*n* = 25) Group II—control group (placebo) (*n* = 25)	Patients advised to brush teeth with propolis toothpaste 3 times/day for 3 min over the course of 35 days.	In group A (used toothpaste with propolis and plant oils) for gingival condition, GBI was significantly decreased for molars *p* = 0.0017, for incisors *p* = 0.007, and total GBI *p* = 0.002.	[91]
			Significant improvement in oral hygiene index (OHI) was observed *p* = 0.011.	
Propolis (Mashhad, Iran)	Total participants (*n* = 40) Two groups: Group I—test group (propolis mouthwash) (*n* = 20) Group II—control group (chlorhexidine mouthwash) (*n* = 20)	Test group was advised to use propolis mouthwash for 3 weeks after brushing their teeth twice/day consecutively.	A statistically significant difference between the score of periodontal index (*p* = 0.005), PI (*p* < 0.001) and GI (*p* = 0.006) in the test group is observed.	[92]

PD—probing depth; CAL—clinical attachment level; FMPS—full month plaque score; FMBS—full month bleeding score; PBI—papillary bleeding index; PME—propolis and mangosteen extract; PM—peri implant mucositis.
